# Dr. William West

**Published:** 1838-01

**Authors:** 


### DR. WILLIAM WEST, OF DUBLIN.

AT Dublin, on the 8th of October, 1837, William West, M.D., in the thirty-fourth year of his age. In recording the premature death of this amiable and excellent gentleman, we bear a willing testimony to his great talents and acquirements as a scholar and physician. He was one of the most esteemed of our contributors, and our counsellor and assistant in the difficulties of some of the northern languages. We borrow the following short notice of him from the *Dublin University Magazine*, for November, 1837.

"As a student of modern languages, Dr. West's success was, in Ireland at least, unrivalled. His accurate and familiar acquaintance with the most intricate and least-studied dialects of northern Europe had earned for him a reputation as a linguist which was not confined to the circle of his native shores. By many learned men on the continent his opinions were sought after with anxiety, and attended to with respect. We have a melancholy satisfaction in acknowledging the obligations under which he frequently placed this Journal, by contributions, the place of which it is impossible to supply. Every day was adding alike to his knowledge and his reputation, when his distinguished career was brought to a close in an early grave."

In the university, Dr. West had borne the highest honours of the under-graduate course. At a later period of his life, the Royal Irish Academy conferred on him the high distinction of electing him a member of their council. In private life, universally respected and esteemed, few men had more of warmer friends; none, perhaps, ever made fewer enemies. The daily press, of all parties, has borne honorable and abundant testimony to the general regret which was occasioned by his premature decease. From the many tributes to his memory which the melancholy event called forth, we select the following from the columns of the *Dublin Evening Mail:*

"When we consider the estimation in which this individual was held as a sound and erudite scholar by the most learned men of the day, the ardent zeal with which he prosecuted science, and the premature age (only thirty-four) at which he was cut off, it is not too much to say that his loss was a public one, and one that must be deeply deplored by the world of letters. The promises held out by his collegiate success were more than realized in the distinction his labours in medicine, philology, geography, and botany subsequently acquired for him. Of these subjects, the science of language occupied his especial attention; and so accurate and profound was his knowledge in this branch of literature, that in matters of abstruse research his assistance was urgently sought after by the most eminent philologists, both at home and abroad. His capabilities in this respect were practically and disinterestedly applied to rendering the most recent discoveries in medicine throughout Europe available to his professional brethren at home. The purity of mind, singleness of purpose, and almost childish simplicity of thought in the ordinary affairs of the world, so often the characteristic accompaniments of true genius, were leading features in Dr. West's character. He may be said to have fallen a sacrifice to his too-ardent pursuit of science, as the fatal fever which terminated his short but distinguished career was caused by close application, whilst in a delicate state of health, in preparing for the British Association an elaborate critical illustration, through its primitive dialects, of the ancient geography and history of Gaul and the British Isles."

---

